# Molecular turnover, the H3.3 dilemma and organismal aging (hypothesis)

**DOI:** 10.1111/acel.12332

**Published:** 2015-02-26

**Authors:** Evelyne Saade, Iryna Pirozhkova, Rakhan Aimbetov, Marc Lipinski, Vasily Ogryzko

**Affiliations:** 1 Faculty of Public Health, Lebanese University LUBeirut, Lebanon; 2 Institute Gustave Roussy, University Paris SUD114, rue Edouard Vaillant, Villejuif, 94805, France

**Keywords:** aberrant repair, aneuploidy, chromatin, epigenetic information, Hayflick limit, somatic stem cells

## Abstract

The H3.3 histone variant has been a subject of increasing interest in the field of chromatin studies due to its two distinguishing features. First, its incorporation into chromatin is replication independent unlike the replication-coupled deposition of its canonical counterparts H3.1/2. Second, H3.3 has been consistently associated with an active state of chromatin. In accordance, this histone variant should be expected to be causally involved in the regulation of gene expression, or more generally, its incorporation should have downstream consequences for the structure and function of chromatin. This, however, leads to an apparent paradox: In cells that slowly replicate in the organism, H3.3 will accumulate with time, opening the way to aberrant effects on heterochromatin. Here, we review the indications that H3.3 is expected both to be incorporated in the heterochromatin of slowly replicating cells and to retain its functional downstream effects. Implications for organismal aging are discussed.

## Organismal vs. replicative aging and epigenetic vs. genetic information

Many mechanistic studies of aging performed at both the molecular and cellular levels focus on the understanding of cellular (or replicative) senescence, that is, the loss of proliferative potential in long-term cultures of primary cells. This phenomenon was first discovered when cells were passed in culture until, after approximately 50 cumulative population doublings (CPDs), they could not be further stimulated to proliferate and reached the so-called Hayflick limit (Hayflick, 1985). Premature induction of irreversible growth arrest and other marks of cellular senescence [including specific cell morphology, senescence-associated beta-galactosidase (SABG), and senescence-associated heterochromatin foci (SAHFs)] (Bayreuther *et al*., 1988; Dimri *et al*., 1995; Campisi & d'Adda di Fagagna, 2007) can be provoked by various stress inducers (Brack *et al*., 2000), including oncogenic stress (Serrano *et al*., 1997), oxidative stress (Horikoshi *et al*., 1986; Ogryzko *et al*., 1996), and even inhibitors of histone deacetylases (HDAC) (Ogryzko *et al*., 1996).

For clarification, cellular senescence should be distinguished from the true subject of gerontology – organismal senescence – ‘a progressive deterioration of physiological function, an intrinsic age-related process of loss of viability and increase in vulnerability’ (de Magalhaes, 2004; Campisi, 2005; Jeyapalan & Sedivy, 2008). On the one hand, the contribution of cellular senescence to organismal aging appears rather straightforward, as the loss of proliferative potential can be directly linked to diminished capacity for tissue regeneration, decreased immune response (Effros, 1996), and a deteriorating endocrine system (Gosden, 1996). On the other hand, the existence of aging-related diseases such as Alzheimer's disease and age-related macular degeneration, the increased incidence and morbidity of cardiovascular, autoimmune, and oncological pathologies, as well as the accumulation of birth defects in the progeny of aging individuals together illustrate that not all aspects of aging can be easily explained solely on the basis of a loss of cellular proliferative potential.

In this respect, we propose to focus on cells that either do not replicate in adults or accomplish very few divisions during the lifespan of an organism – that is, far less than set by the Hayflick limit. For the purpose of this review, we will term these cells below Hayflick limit (BHL) cells. Below Hayflick limit cells include postmitotic cells such as terminally differentiated neurons and muscle cells, and female ova, which are formed during embryonic development and remain in a nonproliferating state for decades (Macklon & Fauser, 1999). Adult stem cells in their dormant stage can also be included since for decades before initiating proliferation, they might not enter into division (Orford & Scadden, 2008; Sottocornola & Lo Celso, 2012), as well as cells (e.g., some liver, kidney, and stomach cells) that enter the G_0_ phase semi-permanently after differentiation. Below Hayflick limit cells are interesting for the following reason: On the one hand, they are far from entering the replicative senescence state; on the other hand, due to the constant molecular turnover and active metabolism in these cells (even in the absence of replication), the lifespan of an adult organism should lead to accumulation of irreversible changes, which could contribute to organismal aging.

A second important clarification concerns the nature of the molecular clock that counts the age of a cell (or an organism). In accordance with the genocentric view, which has dominated the biology field until the end of the twentieth century and which considers DNA as the only source of stable information determining cell phenotype, it was natural to expect that the ticks and tocks of the clock are ultimately of a genetic nature; that is, they will somehow reshape the genetic molecular code script through real changes in the genome, for example, telomere shortening (Olovnikov, 1973; Wright & Shay, 2001) or mutations (Vijg & Dolle, 2002), or at least through accumulating DNA lesions due, for example, to oxidative damage (Gensler & Bernstein, 1981; Hoeijmakers, 2009). With the recent surge in epigenetic research, a greater interest has emerged in cellular changes that (i) have a nongenetic nature and (ii) are sufficiently stable to irreversibly accumulate in cells and contribute to the phenomenon of aging.

Epigenetics focuses on the mechanisms of processing of epigenetic information – defined here as information that is both (i) necessary to determine the state of an organism in addition to its DNA sequence (i.e., genetic information) and (ii) relatively stable compared to the characteristic times of metabolic changes and cellular lifespan (Russo *et al*., 1996; Bird, 2007). These mechanisms play a role in the maintenance of differentiated phenotypes in cell lineages during embryonic development and in adult ages, although their primary evolutionary role might have been to protect genetic information, for example, via suppression of parasitic genetic elements (Saade & Ogryzko, 2014).

One principal carrier of epigenetic information is chromatin – a hierarchically organized complex of DNA, histones, and nonhistone proteins (Bernstein *et al*., 2007). Not surprisingly, the recent interest in ‘all things epigenetic’ begat new ideas on the role of chromatin in aging. It has been known since the 60s that DNA methylation is progressively lost with aging (Pogribny & Vanyushin, 2010). With the more recent works of Guarante in yeast (Kaeberlein *et al*., 1999; Guarente, 2000; Lin *et al*., 2000), the discovery of the role of sirtuins and the effects of reservatrol (Howitz *et al*., 2003; Kaeberlein *et al*., 2005), the role of chromatin in the aging process has become a more fashionable field of research (Chatterjee & Williams, 1962; Dimauro & David, 2009; Pegoraro & Misteli, 2009; Feser & Tyler, 2011; McCord *et al*., 2013). So far, however, most of the mechanistic studies have focused on cellular senescence; that is, they have been concerned with how irreversible changes in chromatin could account for the loss of cellular proliferative potential. For example, the group of Bruce Howard suggested the existence of cellular checkpoint mechanisms that monitor the proper maintenance of heterochromatin domains during cell proliferation, and further proposed that defects in their maintenance could contribute to the phenomenon of replicative senescence (Howard, 1996; Ogryzko *et al*., 1996). This early idea is consistent with a more recent observation of the large-scale unraveling of peri/centromeric satellite chromatin (senescence-associated distension of satellites, or SADS), which could manifest the loss of proper maintenance of heterochromatin in aging cells (Cruickshanks *et al*., 2013; De Cecco *et al*., 2013a,b; Swanson *et al*., 2013).

Now, could epigenetic changes also irreversibly accumulate with time in BHL cells thus contributing to organismal, but not replicative, senescence? At first, one might shy away from the idea as, by current definition, epigenetic changes have to be heritable. Accordingly, one should not even formulate the question as the notion of heritability cannot apply to nonproliferating (postmitotic) cells. To deal with this terminological obstacle, the more general term of ‘epigenetic stability’ (i.e., preservation of stable traits regardless of whether cells proliferate or not) can be used to take into account that epigenetic mechanisms (either chromatin based or other) are most likely also involved in long-term preservation of phenotypic traits in nonreplicating cells (Ogryzko, 2008). With this slight adjustment in the scope of epigenetics, we can legitimately ask whether epigenetic factors and/or changes can affect the properties of nonproliferating cells.

In this minireview, we discuss the possibility that in nonreplicating cells, epigenetic modifications, and more specifically very particular changes in chromatin structure – the gradual replacement of canonical histones H3.1/H3.2 with variant histone H3.3 – could contribute to organismal aging by inducing aberrations in gene regulation and other functions in BHL cells. Although this hypothesis has not been directly supported by a plethora of experimental data as yet, the aggregation of existing claims and accumulating evidence leads almost inevitably to paradoxical conclusions about the role of H3.3 in BHL cells with tempting implications with regard to the aging process. The ‘H3.3 dilemma’, as we term this situation in the field, is both sufficiently intriguing and convincing to be worth-raising, in the hope that it will trigger new directions and efforts for research.

## Alternative histones in general and H3.3 in particular

Alternative histone variants (replacement histones) are the latest addition to the growing list of potential epigenetic marks carried by chromatin, which also include DNA methylation and histone post-translational modifications. Although discovered a long time ago, these variants have attracted renewed interest in the last 10 years with the recognition of their various roles in genome function (Luger *et al*., 2012). Thus, their presence was found to correlate with particular functional states of chromatin (Table1); for example, histone macroH2A is enriched in silenced chromatin, whereas H2A.BBD is associated with euchromatin and splicing/RNA processing (Costanzi & Pehrson, 1998; Chadwick & Willard, 2001a,b).

**Table 1:** Core histone variants, their functions, and features

Histone	Biological function and features	Conservation	References
H3 variants			
H3.3	Gene activation, silencing, and chromosome segregation. Can be deposited in replication-independent way	Yes, but in yeast, it is the only noncentromeric H3 variant	Ahmad & Henikoff (2002), Elsaesser *et al*. (2010), Filipescu *et al*. (2013)
CenpA	Epigenetic marker of centromere	Present in most of eukaryotes, but less conserved than other H3 histones	Palmer *et al*. (1991), Cleveland *et al*. (2003), Bailey *et al*. (2013)
H3.X	Euchromatin in primates	Primate specific	Wiedemann *et al*. (2010)
H3.4/H3t	Sperm genome and nucleolus of somatic cells	Mammalian specific	Tachiwana *et al*. (2010)
H3.Y	Euchromatin in primates	Primate specific	Wiedemann *et al*. (2010)
H3.5/H3.3c	Euchromatin in hominid testis	Hominid specific	Schenk *et al*. (2011)
H2A variants			
H2AZ	Poising genes for activation. Gene activation, gene silencing, and chromosome segregation	Present in most of eukaryotes	Faast *et al*. (2001), Creyghton *et al*. (2008), Eirin-Lopez *et al*. (2009), Hu *et al*. (2013)
macroH2A	Association with repressed/silenced chromatin, large size due to an additional C-terminal domain	Vertebrate specific	Costanzi & Pehrson (1998), Buschbeck *et al*. (2009), Gamble *et al*. (2010)
H2A.BBD	Splicing, replication Active transcription	Mammalian specific	Ioudinkova *et al*. (2012), Tolstorukov *et al*. (2012)
H2AX	Double-strand break repair/meiotic remodeling of sex chromosomes and genome integrity. The function is mediated by the phosphorylated form γH2A.X	Present in most of eukaryotes	Fernandez-Capetillo *et al*. (2003), Sedelnikova *et al*. (2003)
H2B variants			
TH2B	Chromatin to nucleoprotamine transition		Gineitis *et al*. (2000), Li *et al*. (2005), Govin *et al*. (2007), Montellier *et al*. (2013)
H2BFWT	Sperm telomere binding	Primate specific	Gineitis *et al*. (2000), Churikov *et al*. (2004)
H2BE	Transcription regulation in olfactory neurons		Santoro & Dulac (2012)

A striking example for an epigenetic role of replacement histones is that of CenpA, an H3 variant that has been shown to serve as a self-perpetuating mark on chromatin, important in the maintenance and reproduction of centromere chromatin regardless of its underlying DNA sequence (Cleveland *et al*., 2003; Bailey *et al*., 2013). As another distinct feature, the sequence of CenpA varies significantly between homologs in different species, which might reflect the role of improper chromosome segregation (and resulting meiotic incompatibility) in speciation (Henikoff *et al*., 2004; Probst *et al*., 2009).

Our main focus here concentrates on another H3 variant – the alternative histone H3.3. Unlike CenpA, H3.3 is conserved through a wide range of species and differs by only few amino acids from its canonical counterparts H3.1 and H3.2 (4 and 5 replacements, respectively, mostly in the ‘AAIG’ vs. ‘SAVM’ patch at aa 87–90 in the histone sequence) (Elsaesser *et al*., 2010; Filipescu *et al*., 2013). Despite their relative modesty, these changes have two dramatic consequences.

First, unlike the replication-coupled (RC) deposition of its canonical counterparts into chromatin, the deposition of H3.3 is replication independent (RI) (Ahmad & Henikoff, 2002). Whereas H3.1/2 copurify with the histone chaperone CAF-1, which is responsible for their RC deposition, there are different RI pathways for H3.3 incorporation into chromatin. HIRA (Lamour *et al*., 1995) is the chaperone complex that is responsible for H3.3 deposition at actively transcribed regions (Tagami *et al*., 2004; Goldberg *et al*., 2010), whereas the DAXX–ATRX complex (Drane *et al*., 2010; Goldberg *et al*., 2010) and DEK (Sawatsubashi *et al*., 2010) can deposit H3.3 into heterochromatin and regulatory regions, including that of intermediate response genes in neurons (Michod *et al*., 2012). Interestingly, changing any one of the residues specific to H3.1/2 to those present in H3.3 relieves the block to RI assembly and allows histone H3 deposition outside of S phase (Ahmad & Henikoff, 2002), suggesting that RI deposition is the default pathway and that H3.1/2 are actively recognized and blocked from RI deposition. That in the yeast *Saccharomyces cerevisiae*, the only H3 histone involved in regular chromatin structure is similar to H3.3, reinforces the notion that H3.3 assembly is the default H3 deposition pathway. Consistently, it was shown that unlike H3.3, H3.1 colocalizes with replication sites (Ray-Gallet *et al*., 2011). The same study showed that H3.3 can be deposited at replication sites when H3.1 deposition is impaired, but that the opposite is not true: H3.1 cannot replace H3.3 when the incorporation of the latter is affected.

As another remarkable feature, H3.3 is associated with actively transcribed chromatin. Several lines of evidence established this correlation. In 1984, H3S, an H3.3-like histone, was found in ciliates only in the active macronucleus, whereas the canonical H3-like histone H3F was found in the transcriptionally inactive micronucleus (Allis & Wiggins, 1984). Two decades later, H3.3 deposition in *Drosophila* was shown to localize to active rDNA arrays and euchromatin but not heterochromatin (Ahmad & Henikoff, 2002). Working on human cells, Janicki *et al*. (2004) showed that H3.3 deposition took place on a transgene array whose transcription was activated.

The association of H3.3 with active chromatin is supported by the analysis of histone post-translational modifications (PTMs). Mass spectrometry analysis of *Drosophila* H3.3 shows that it is enriched in active chromatin marks (methylation at K4 and K79 and acetylation at K9, K14, and K18 + K23) and that it has lost PTMs’ characteristic of repressed chromatin such as dimethyl lysine 9 (McKittrick *et al*., 2004; Mito *et al*., 2005). In mammalian cells, the majority of modifications detected on H3.3 are also marks of active chromatin, including methylation of K4 and K79 and acetylation of K14, K18, and K23 (Hake & Allis, 2006).

Finally, using chromatin immunoprecipitation (ChIP) technology, H3.3 was shown specifically incorporated throughout the gene body of transcribed genes and highly enriched in promoter regions in both *Drosophila* and mammalian cells, its presence correlating with that of bound RNA polymerase II (Janicki *et al*., 2004; Chow *et al*., 2005; Wirbelauer *et al*., 2005; Daury *et al*., 2006; Mito *et al*., 2007; Nakayama *et al*., 2007; Jin *et al*., 2009; Sutcliffe *et al*., 2009; Tamura *et al*., 2009).

## H3.3 – a transcription player or a ‘placeholder dummy’? The ‘H3.3 dilemma’

**Fig 1:**
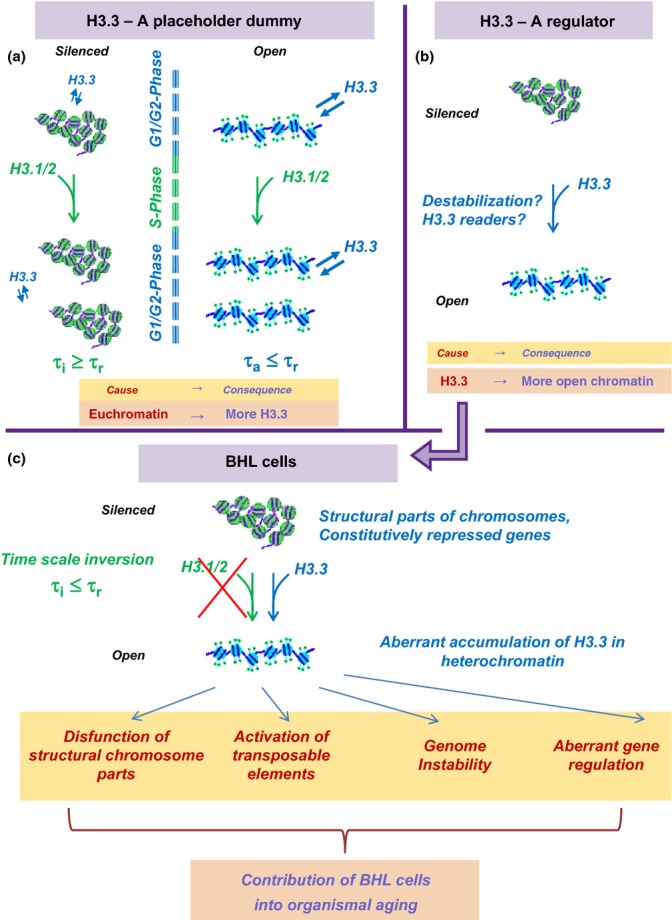
H3.3 Dilemma. *Top*. Alternative explanations for association of H3.3 with active chromatin. (a) A placeholder dummy. Euchromatin (right) is more open and more prone to histone damage and exchange than heterochromatin (left). Time of turnover of H3 histones in active chromatin *τ*_a_ is shorter than time of cell replication *τ*_r_. Accordingly, replication-coupled deposition of canonical H3.1/2 cannot be responsible for the turnover of all H3 histones, which is expected to lead to a preferential accumulation of H3.3 in open chromatin. To the contrary, the turnover rate of H3 in heterochromatin *τ*_i_ is slower than *τ*_r_, and the replication-coupled H3.1/2 deposition can be sufficient for H3 replacement. (b) A regulator. H3.3-containing chromatin is ‘special’ in some respect, and H3.3 replacement of the canonical H3.1/2 leads to chromatin opening or other consequences for its structure and function. *Down*. Consequences of the ‘regulator’ model in the case of below Hayflick limit (BHL) cells. The replication time of BHL cells is expected to be slower than the rate of H3 exchange in heterochromatin, which should lead to an accumulation of H3.3 histones in heterochromatin of BHL cells with potentially negative consequences in terms of structure and function.

The simplest possibility (the ‘zero hypothesis’) to account for the association of H3.3 with active chromatin derives from the facts that (i) active chromatin is more open, therefore more dynamic and more prone to molecular turnover and (ii) the deposition of canonical H3.1/2 histone is strictly replication-coupled. Let us designate *τ*_a_ the characteristic time of H3 histone turnover at active chromatin sites, *τ*_i_ the analogous time for inactive chromatin, and *τ*_r_ the characteristic time of cell replication. Photobleaching experiments indicate that *τ*_i_ ≥ *τ*_r_ for typical cells in culture (Kimura & Cook, 2001), whereas *τ*_a_ could be significantly shorter than *τ*_r_. Accordingly, for active states of chromatin, the deposition of canonical H3 forms is too slow (∼*τ*_r_) to catch up with the molecular turnover, the only alternative being in the deposition of H3.3 leading to its accumulation at corresponding sites. Thus, in the framework of the ‘zero hypothesis’, H3.3 is only a ‘placeholder dummy’ that replaces H3.1/2 in a nonreplicative context. Its association with active genes is therefore nothing more than a downstream consequence of an open state of chromatin (Fig.1a).

A more attractive possibility has it that the H3.3 variant is causally involved in the establishment of an active/open chromatin state (Fig.1b). There are many ways whereby the replacement of H3.1/2 by H3.3 could affect chromatin, including changes in nucleosome stability or creation of docking sites for regulatory proteins (i.e., ‘H3.3 readers’), etc. Understandably, the idea that H3.3 is a player in gene regulation is a more stimulating hypothesis. The notion that H3.3-containing nucleosomes are in some way ‘special’ serves as a direct motivation for the hypothesis of their semiconservative replication (Nakatani *et al*., 2004; Jin *et al*., 2009). It is also more consistent with the mechanistic roles of other histone replacement variants such as CenpA, macroH2A, and H2AZ, in the establishment of particular functional chromatin states (Bonisch & Hake, 2012; Skene & Henikoff, 2013). However, the differences between these histone variants and their canonical counterparts are more significant than the 4(5) aminoacid difference between H3.3 and H3.1/2. This calls for more rigor in establishing the possible downstream effects of H3.3 presence. In addition, the fact that H3.1/2 deposition on chromatin requires replication sets a constraint on the plausible properties of H3.3 histone, which could be formulated as a dilemma that emerges when considering two features of H3.3 deposition in BHL cells (Fig.1c).

### Feature 1. *τ*_r_* *≥ *τ*_i_ in BHL cells

In an adult organism, BHL cells are expected to accumulate H3.3 in their heterochromatin. Indeed, due to their slow replication time, they cannot incorporate the replication-coupled canonical H3.1/2 histones at a speed sufficient to compensate for H3 molecular turnover in all chromatin types.

There are many causes for molecular turnover of histones, including thermal fluctuations, oxidation, proteolysis (Adams-Cioaba *et al*., 2011) and active chromatin remodeling due to DNA repair, all events expected to regularly necessitate the incorporation of new histone molecules in chromatin. Whereas in actively replicating cells *τ*_i_ ≥ *τ*_r_, that is, the rate of canonical H3 deposition should be sufficient to compensate for the loss of H3 in inactive chromatin, BHL cells live for decades with, at best, little replication. The decades-long time scales are not comparable with the rate of molecular turnover of proteins in a living cell. This is, essentially, the same problem that motivated Francis Crick to propose his ‘epigenetic templating’ model of long-term synaptic potentiation in neurobiological memory (Crick, 1984; Ogryzko, 2008).

Exacerbating the problem, H3 is the only core histone with cysteine residues and thus should be more sensitive to oxidation than other histones. Moreover, without timely replacement of a histone molecule, this oxidation will progress irreversibly: Unlike disulfide forms, RS-SR which can be reduced back to thiol groups RSH, sulfenic (RSO^−^), sulfinic (RSO_2_^−^), and sulfonic (RSO_3_^−^) forms of cysteine cannot be restored.

With the inverted relation *τ*_r_ ≥ *τ*_i_ in BHL cells, H3 would eventually require a replacement at all sites in the genome, thus including repressed chromatin. Unlike the replication-coupled H3.1/2 deposition, the H3.3 RI pathway remains available at all times in BHL cells; hence, one should expect a gradual substitution of H3.1/2 by H3.3 in repressed chromatin.

### Feature 2. Aberrant effects on repressed chromatin

Now, let us consider that the association of H3.3 with active chromatin is due to its role in establishing an active and open chromatin state. In this case, one should expect that its incorporation into heterochromatin could affect BHL cells in an undesirable way. Heterochromatin-based silencing is an essential mechanism employed to restrict gene expression to housekeeping and lineage-specific genes, as well as to suppress parasitic selfish elements (e.g., transposons). Repetitive sequences with a structural role (such as satellite DNA) need also be transcriptionally silenced. One can see how the opening of otherwise silenced chromatin in inappropriate contexts would lead to unwanted transcriptional activation (or else competition with other genomic sites for binding of available transcription factors) with negative consequences due to perturbed epigenetic programs and induction of genome instability (via activation of transposable elements and/or affecting structural parts of the chromosomes).

Accordingly (unless we are prepared to consider the consequences for organismal aging discussed below), we are faced with ‘the H3.3 dilemma’: (i) either H3.3 is a mundane ‘placeholder dummy’; that is, H3.3-containing nucleosomes are not ‘special’ and are tolerable in any amounts at any place in the genome, (ii) or H3.3 is not incorporated in BHL cells at the most inappropriate genome locations, perhaps because in these cells, chromatin deposition of H3.1/2 is not as strictly replication-coupled as imagined and thus occurs at a low albeit sufficient rate.

At this point, it is worth-noting that the DAXX-ATRX chaperone system has been shown to facilitate H3.3 deposition in many nongenic repeat regions of the genome (Filipescu *et al*., 2013). Consistent with this fact, recent studies indicate that the notion of H3.3 associated only with gene activation is a clear oversimplification. Instead, the emerging view implicates H3.3 in the establishment of a chromatin landscape which would allow proper gene expression upon cell differentiation, for example the bivalent chromatin landscape in embryonic stem cells (Banaszynski *et al*., 2013). Still, despite its nuances, this view remains consistent with the notion of H3.3 incorporation affecting chromatin properties and function. Thus, the problem persists of the downstream effects of a H3.3 presence at inappropriate sites of the genome in BHL cells.

Unlike H3 histones, the H2A/H2B histones and their variants are subject to a relatively fast exchange (at a time scale less than the replication time of a cell) and their deposition is not coupled to DNA replication. Two distinct features pertain to H3 histones: (i) the difference between the RC and RI pathways and (ii) their relatively slow rate of exchange; this is what is responsible for the H3.3-specific dilemma.

In the next section, we will review how both horns of the dilemma fare with regard to the experimental evidence.

## Horns of the dilemma – experimental observations

### H3 protein is mostly represented by the H3.3 variant in terminally differentiated and quiescent cells

Despite H3 being the only core histone containing cysteine and thus more prone to oxidation, no age-related accumulation of oxidized histone H3 (Carter & Chae, 1975) has been detected, indicating that cells have a way to replace oxidized H3 histones.

In this respect, a recent proteome-wide study that measured protein molecular turnover using stable isotope chase combined with mass spectrometry (Toyama *et al*., 2013) must be discussed. In mammals, most proteins have an average half-life of 1–2 days. Some, however, (e.g., crystallins, nucleoporins) exhibit exceptionally long half-lives up to several months (as judged by the significant presence of heavy isotope-labeled versions of corresponding peptides in 6- or 12-month-old tissues). Strikingly, histone H3.1 has the slowest turnover with only 10% of the protein replaced in 6 months in rat brain tissue. The anomalously high stability of H3.1 would appear to invalidate the main premise of the H3.3 dilemma. However, the steady state assumption used to justify the half-life estimations of protein stability is not valid for the canonical H3 histone. The case of H3.1 molecular turnover is special because in nondividing cells, it is replaced by a different molecule – H3.3. It is thus not surprising that most of the H3.1 present in nonreplicating cells is represented by molecules deposited at a young age, because even if the total levels of H3.1 fall dramatically in aged tissue, the replacement comes not in the form of fresh H3.1 molecules, but as H3.3 histone.

Indeed, H3.3 has been shown to progressively replace most H3.1/2 in terminally differentiated cells in vertebrates. In quiescent human T lymphocytes, for instance, H3.3 is the only H3 variant synthesized and is the major variant by mass (73%) (Wu *et al*., 1983). It also becomes the predominant form in chicken liver and kidney and also represents up to 90% of H3 molecules in terminally differentiated rat neurons (Urban & Zweidler, 1983; Pina & Suau, 1987).

The latter studies have all been performed in model organisms with a typical lifespan of several years. Due to the molecular protein turnover, it should take no more than a year to substitute H3.1/2 with H3.3. In this regard, the predominance of H3.3 should not come as a surprise, but this begs the question of why so much of canonical (replication-coupled) H3 still remains in chromatin in terminally differentiated cells. The same question is even more acute when considering humans who live much longer.

A partial explanation for the remaining canonical H3 is, of course, DNA repair. Virtually every pathway of DNA repair requires DNA synthesis and involves the PCNA molecule which can recruit the H3.1/2 chaperone CAF1 for deposition of H3.1/2 in the absence of replication (e.g., according to the ‘access-repair-restore’ model (Smerdon, 1991)). However, it has recently been reported that for DNA repair, H3.3 can also be deposited by the HIRA chaperone at sites of DNA damage, important for recovery of transcriptional activity (Adam *et al*., 2013) as well as for progression of the DNA replication fork after UV damage (Frey *et al*., 2014), suggesting that even in the case of repair, not all newly deposited H3 histones are canonical H3.1/2. Furthermore, common wisdom (Goodarzi & Jeggo, 2012; Lemaitre & Soutoglou, 2014) has it that heterochromatin represents a barrier for repair machinery. Accordingly, it is an open question of how much the repair-coupled H3.1/2 deposition can contribute to the maintenance of canonical H3.1/2 at heterochromatin loci in BHL cells. It is possible that some alternative yet to be discovered mechanisms of H3.1/2 deposition at heterochromatin loci (whether linked to slow background DNA synthesis or else DNA synthesis independent) take place in terminally differentiated and other BHL cells. Although it would be useful to confirm these data with modern techniques (such as mass spectrometry and Western blotting with H3.3-specific antibodies), it is indeed very likely that the vast majority of H3 histones in these cells are represented by H3.3, with the consequence that in their heterochromatin (the largest part in the genome), H3.1/2 should be substituted by H3.3.

### Experimental evidence for a special nature of the H3.3 nucleosome

The biological role of H3.3 has been the subject of intensive recent research. Adding more urgency to this effort, increasing evidence implicates H3.3 and its chaperones in cancer (Schwartzentruber *et al*., 2012; Behjati *et al*., 2013; Fontebasso *et al*., 2013; Aihara *et al*., 2014; Venneti *et al*., 2014). Most remarkable is the tumor type specificity of the H3.3 mutations that have been detected - whereas K27 and G34 of H3.3 are affected in 31% of childhood brain tumors (Schwartzentruber *et al*., 2012), 95% of chondroblastomas exhibit K36M alterations, and 92% of giant cell tumors of bone have K27 mutated in this protein (Behjati *et al*., 2013). These facts are hard to reconcile with H3.3 being a simple placeholder for canonical H3. Other recent data provide additional support for the notion that replacement of canonical H3.1/2 by H3.3 has downstream effects on chromatin properties and function.

Incidentally, functional knockouts of the H3.3 gene reveal partial lethality in adult *Drosophila* males (Sakai *et al*., 2009) and misregulation of gene activation in mammals (Placek *et al*., 2009; Sakai *et al*., 2009; Tamura *et al*., 2009; Banaszynski *et al*., 2013; Bush *et al*., 2013). This, however, cannot serve as direct evidence for a special nature of H3.3-containing nucleosomes. Instead, it could be argued that the observed gene expression effects could be simply due to nucleosome depletion in the absence of a functional replication-independent histone deposition pathway; in addition, nucleosome depletion could compromise genome stability. Moreover, H3.3 is the only noncentromeric H3 histone in yeast, and it is also not essential for transcription or viability in *Tetrahymena* (Cui *et al*., 2006). This suggests that, in any case, the search for a special role of H3.3-containing nucleosomes would be most productive in higher eukaryotic systems.

Another class of evidence based on the functional knockouts of the H3.3 chaperones HIRA or DAXX (Yang *et al*., 2011; Pchelintsev *et al*., 2013; Soni *et al*., 2014) and on the interaction of these proteins with known transcriptional regulators, such as BRG1 or HP1γ (Kim *et al*., 2011; Pchelintsev *et al*., 2013), also cannot be interpreted straightforwardly in support of the special nature of H3.3 nucleosomes. It could be that these proteins perform additional and independent roles in gene regulation, which still would be consistent with a placeholder role for H3.3. For example, HIRA binds many genomic sites in the absence of UBN1 and ASF1a, its usual partners in H3.3 deposition, and these ‘HIRA-only’ sites are also not enriched in H3.3 (Pchelintsev *et al*., 2013). Concerning DAXX, its nonchaperone functions have been reviewed (Lindsay *et al*., 2008; Salomoni, 2013); in addition to depositing H3.3, it has also been shown recently to be involved in the deposition of CenpA in aberrant locations in the genome (Lacoste *et al*., 2014). Nevertheless, given the mechanistic association of the HIRA chaperone with gene activation (Yang *et al*., 2011; Pchelintsev *et al*., 2013), it remains tempting to speculate that the role of the DAXX in H3.3 deposition in pericentromeric and other nongenic repeat chromatin domains (Morozov *et al*., 2012; Corpet *et al*., 2014) could be to avoid any adverse consequences of the involvement of HIRA chaperone in H3.3 deposition in the case of heterochromatin and other nongenic repeat sequences (Banaszynski *et al*., 2013).

Biophysical studies are more direct in addressing the issue of the special nature of the H3.3-containing chromatin. Albeit somewhat controversial, they indicate subtle effects on nucleosome stability and positioning (Thakar *et al*., 2009) and increased sensitivity of a H3.3-containing nucleosome to salt-dependent disruption, exacerbated in the presence of a H2AZ histone variant within the same nucleosome (Jin *et al*., 2009). On the other hand, the hybrid CenpA/H3.3 nucleosome is unusually stable, an observation that was linked to CenpA mislocalization and resulting chromosome aberrations in cancer (Arimura *et al*., 2014). Another study (Chen *et al*., 2013) points to the higher-order folding of chromatin as the level of chromatin organization where the effects of H3.3 are mostly manifest.

The most convincing evidence for a special nature and/or role of H3.3-containing chromatin would be whether proteins or protein domains were found specialized in distinguishing between H3.1/2 and H3.3 (i.e., ‘H3.3 readers’, consistent with the influential concept of the ‘histone code’ (Hake & Allis, 2006)). Remarkably, three recent papers claim to accomplish Just that, both pointing at the crucial role of aminoacid A-S(T)31 replacement. First, the potential tumor-suppressor protein ZMYND11 has been shown to specifically recognize H3.3, trimethylated at K36, and to curtail RNA polymerase II-driven RNA elongation (Wen *et al*., 2014). Importantly, in addition to K36me3 bound by the ZMYND11 PWWP domain, the H3.3-specific S31 (replaced by alanine in other H3 histones) also contributes to this interaction by being lodged into the bromo-ZnF-PWWP ‘valley’, greatly augmenting the affinity between the two proteins. More recently, a related study revealed the importance of this recognition for regulation of RNA splicing (i.e., intron retention) (Guo *et al*., 2014).

Second, ATXR5/6, a specific histone methyltransferase from *Arabidopsis*, has been shown to do the opposite, specifically targeting the H3.1 variant, ‘reading’ the alanine 31 which is replaced in the H3.3 histone (Jacob *et al*., 2014). The authors proposed a model whereby a specific heterochromatin mark (H3K27me1) is maintained during DNA replication. The important implication is that this mark cannot be maintained after H3.1/2 has been replaced by H3.3 (due to the inability of ATXR5/6 to methylate H3.3), which could contribute to the opening of a previously inactive chromatin, and be responsible for, for example, the induction of transposable elements.

## H3.3 dilemma and aging – organismal and replicative

Let us now return to the H3.3 dilemma. The experimental evidence strongly suggests that H3.3 nucleosomes are both (i) ‘special’ and (ii) do eventually replace H3.1/2 in the heterochromatin of BHL cells. Accordingly, as far as BHL cells are concerned, it cannot be ‘business as usual’ and there are obvious implications for organismal aging.

We are far from proposing that H3.3 accumulation in BHL cells would provide a unifying theory of organismal aging, a multifaceted phenomenon that cannot be reduced to one universal cause. The question we ask, however, is whether some aspects of organismal aging could be due to the eventual accumulation of H3.3 in heterochromatin of BHL cells leading to aberrations in gene expression and genome instability. Various experimental models can be examined in this respect, pertaining to diverse aspects of an aging organism.

### Aging females

**Fig 2:**
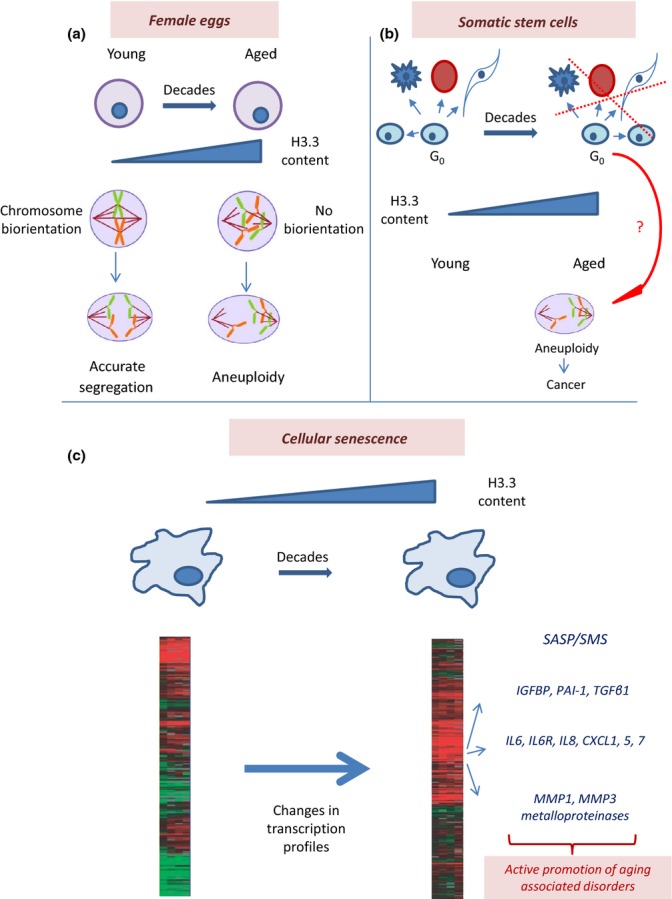
Possible relationship between the H3.3 dilemma and organismal aging. (a) Aging eggs might accumulate H3.3 in structural parts of their chromosomes, leading to negative consequences in chromosome/chromatid cohesion, resulting in increased aneuploidy. (b) Somatic stem cells might accumulate H3.3 in their heterochromatin, which could result in epigenetic reprogramming and negative consequences in terms of differentiation and self-renewal properties. Also, aneuploidy might contribute to increased cancer frequency in aged individuals. (c) In senescent cells, a Misincorporation of H3.3 at inappropriate genome locations could lead to changes in transcription profiles responsible for the specific (e.g., proinflammatory) properties of senescent cells (SASP/SMS phenomenon), which could actively contribute to organismal aging.

With age, fertility decreases, the number of miscarriages increases as well as the frequency of congenital birth defects in the newborns (Gosden, 1985; Hassold & Chiu, 1985; Stein, 1985; Gindoff & Jewelewicz, 1986; Piette *et al*., 1990) (Fig.2a). The most prominent factor is aneuploidy (e.g., strikingly increasing trisomy 21), indicating that ova quality decreases with age. Given that germline proliferation in the ovary terminates during fetal development, women's eggs have to remain in a nonproliferative state for decades and thus should qualify as *bona fide* BHL cells subject to protein turnover and H3.3 accumulation in heterochromatin. Intriguingly, although the causes of age-related aneuploidy (Angell, 1997; Lamb *et al*., 1997; Wolstenholme & Angell, 2000) are still under debate, the loss of cohesion between homologous chromosomes or chromatids which produce segregation errors appears to be an important mechanism (Wolstenholme & Angell, 2000; Schramm *et al*., 2002; Pellestor, 2004; Pellestor *et al*., 2006). Investigations in mice suggest that a loss of cohesin complex could be responsible (Chiang *et al*., 2010; Lister *et al*., 2010), but in humans (Garcia-Cruz *et al*., 2010) no differences are observed in the levels of meiotic cohesins in oocytes of different ages, indicating that loss of cohesin cannot be the only cause for chromosome nondisjunction in the eggs of aging females. It is obviously tempting to speculate that an improper accumulation of H3.3 in specific structural parts of chromosomes (such as centromeric and pericentromeric chromatin, which are heterochromatic) could affect cohesion and other properties, thereby contributing to meiotic defects and aneuploidy.

### Adult/somatic stem cells

Another particular class of BHL cells is represented by adult (somatic) stem cells (SC) (Fig.2b). An important and relevant feature is their ability to stay in a quiescent state for decades (Orford & Scadden, 2008; Li & Bhatia, 2011; Sottocornola & Lo Celso, 2012) before being induced to proliferate and/or differentiate. This property has been linked to their long-term reconstituting capacity (Reya *et al*., 2001). Defects in regulation of quiescence can lead to premature exhaustion of the SC pool causing failure in tissue regeneration (Cheshier *et al*., 1999; Arai *et al*., 2004) needed, for example, following myelotoxic insults (Cheshier *et al*., 1999). Thus, the H3.3 dilemma considerations do pertain to the biology of adult SC, especially during their nonreplicating stage, even though they are little affected by the Hayflick limit. Despite many technical challenges in isolating and working with dormant adult SC, future research should shed light on how the accumulation of H3.3 in heterochromatin could affect their main characteristics: capacity to self-renew and differentiate, which is directly relevant to organismal aging. Furthermore, chromosome cohesion defects and the resulting aneuploidy upon exiting a long-term dormant stage could have oncological implications. An additional open question – which could be seen as an offshoot of the H3.3 dilemma when applied to adult SC – is whether a high H3.3 content could serve as a marker for dormant somatic SC.

### Cellular senescence (Fig.2c)

Up till now, we have restricted our discussion to the possible role of BHL cells in organismal aging, intentionally excluding the phenomenon of cellular senescence. In fact, H3.3 has been previously discussed in such a context (Rai & Adams, 2012), and more recent data suggest that this variant histone and its proteolytically processed form could drive cellular senescence (Duarte *et al*., 2014), most likely through stress-induced mechanisms.

This section, however, focuses on how H3.3 can be relevant for a different aspect of the biology of senescent cells – that is, for understanding the postmitotic stage of cellular senescence and its contribution to organismal aging. Indeed, given that cells can be growth-arrested for decades, the logic behind molecular turnover and H3.3 accumulation should apply to the cellular senescent state as well. One can ask whether some distinguishing characteristics of senescent cells could relate to the accumulation of H3.3 and consequent aberrations in chromatin function. For example, the above-mentioned SADS phenomenon (Cruickshanks *et al*., 2013; De Cecco *et al*., 2013a,b; Swanson *et al*., 2013) could be a manifestation of improper incorporation of H3.3 in the absence of replication – that is, it might be not the upstream cause of growth arrest (the suggestion consistent with the Howard hypothesis (Howard, 1996; Ogryzko *et al*., 1996)), but rather a downstream consequence thereof.

More importantly, changes in the gene expression profiles due to H3.3 misincorporation can help to explain how cellular senescence contribute to organismal aging – in ways that come in addition to the mere loss of proliferation potential and limited tissue regeneration. One of the marks of cellular senescence is senescence-associated secretory phenotype (SASP), which results in the secretion of various growth factors, cytokines, and proteases (called summarily senescence messaging secretome (SMS) (Kuilman & Peeper, 2009)), leading to age-related tissue dysfunction and disruption (Coppe *et al*., 2008; Rodier & Campisi, 2011). The SMS includes IGFBP, PAI-1, TGFβ1 (Tremain *et al*., 2000; Kortlever *et al*., 2006; Wajapeyee *et al*., 2008), and immune regulators such as IL6, IL6R, IL8, CXCL1, 5, and 7 (Shelton *et al*., 1999; de Magalhaes *et al*., 2004; Xue *et al*., 2007; Acosta *et al*., 2008; Kuilman *et al*., 2008), and secretion of proinflammatory proteins by senescent cells may be involved in positive feedback loops inducing senescence in neighbor cells (Acosta *et al*., 2008; Kuilman *et al*., 2008; Freund *et al*., 2010). Also, the activity of the metalloproteinases MMP1 and MMP3 which degrade the extracellular matrix is increased in senescent cells (Krizhanovsky *et al*., 2008). Most importantly, it has been shown that clearance of senescent cells delays aging-associated disorders in mice (Baker *et al*., 2011), strongly supporting the notion that the presence of senescent cells actively promotes (presumably, via SASP/SMS) these disorders, cancer included (Krtolica *et al*., 2001). A crucial question is how changes occur in the transcriptional program during cellular senescence. A tempting, although still speculative, explanation is provided by the H3.3 dilemma – misincorporation of H3.3 into the otherwise suppressed chromatin of senescent cells could lead to their reprogramming and aberrant gene expression, with SASP an eventual (although not necessarily direct) result of such changes. Consistent with this idea, a recent study from the Adams group demonstrates that the HIRA chaperone plays an important role in chromatin dynamics in senescent cells and is responsible for the changes in gene expression profiles (Rai *et al*., 2014).

## Conclusion. ‘Aberrant Chromatin repair’?

Natural wear and tear of chromatin is unavoidable during the decades-long lifespan of BHL cells. With chromatin considered the principal carrier of epigenetic information, an important question arises as to the way its histone components are reconstituted. H3 presents a special challenge in this regard. On the one hand, it is buried deep inside the nucleosome and cannot be readily replaced by passive off-and-on molecular exchanges. On the other hand, the presence of cysteine residues makes it more prone to oxidative stress. In most eukaryotes, evolution has chosen a way, somewhat similar to two alternative strategies of dealing with DNA damage: Whereas replication-coupled deposition of H3.1/2 corresponds to passive removal of a ‘lesion’ via its dilution among the exponentially growing number of normal copies, replication-independent H3.3 deposition is an active replacement, corresponding to, for example, the DNA nucleotide excision repair or base excision repair pathways. Consistent with the notion that epigenetic information can be damaged and thus needs be repaired, RI H3.3 deposition could be considered a specialized ‘epigenetic repair pathway’.

Recent evidence, however, indicates that H3.3 is not a simple doppelganger of H3.1/2, but, at least in higher eukaryotes, has its own personality, a unique voice in the epigenetic cellular orchestra. If this is not a placeholder dummy and the information content of chromatin is changed after H3.1/2 replacement, a more apt analogy would be an aberrant DNA repair (Kim *et al*., 2014; Talhaoui *et al*., 2014). Here, we have considered some constraints that the properties of H3.1/2 and H3.3 set on plausible models of H3.3 function and we have discussed the potential implications of ‘aberrant chromatin repair’ for organismal aging, with some tackling of the properties of senescent cells. We believe that these considerations, while admittedly speculative at the moment, have a potential to trigger further research.

We see another angle where DNA repair could be relevant in the context of the H3.3 dilemma. DNA lesions are repaired less efficiently in heterochromatin, in part because the effects of such lesions are not expected to manifest themselves in silenced genes, repair being thus less necessary (Goodarzi & Jeggo, 2012; Lorat *et al*., 2012). Improper H3.3 accumulation in heterochromatin could activate otherwise silenced genes and synergize with the negative effect of unrepaired DNA lesions which accumulate therein. This is another facet of the H3.3 dilemma that awaits further investigation.

We have provided a number of arguments to suggest that the H3.3 replacement histone could play an important role in organismal and cellular aging via improper incorporation into the heterochromatin of BHL cells. Accordingly, the ability to manipulate H3.3 deposition and/or its downstream effects might open a new way for epigenetic treatment and prophylaxis of aging.
